# Harmol used for the treatment of herpes simplex virus induced keratitis

**DOI:** 10.1186/s12985-024-02384-0

**Published:** 2024-05-27

**Authors:** Huanhuan Xu, Nan Zhou, Zhenping Huang, Jing Wu, Yajie Qian

**Affiliations:** 1 Department of Ophthalmology, Jinling Hospital, Nanjing Medical University, Nanjing, Jiangsu China; 2 Department of Caries and Endodontics, Nanjing Stomatological Hospital, Medical School of Nanjing University, 30# Zhongyang Road, Xuanwu District, Nanjing, Jiangsu 210008 China; 3 Medical School of Nanjing University, 22# Hankou Road, Nanjing, 210093 Jiangsu Province China; 4 Department of Ophthalmology, Affiliated Drum Tower Hospital, Medical School of Nanjing University, 22# Hankou Road, Nanjing, Jiangsu 210093 China

**Keywords:** Herpes simplex virus type 1, Herpes simplex keratitis, ACV-resistance, Harmol

## Abstract

Herpes simplex virus type 1 (HSV-1) infection of the eyes results in herpes simplex keratitis (HSK), which has led to vision loss and even blindness in patients. However, the rate of drug resistance in HSV is on the rise; therefore, new antiviral agents with sufficient safety profiles must be developed. At present, we assessed the anti-HSV-1 activity of 502 natural compounds and their ability to reduce the HSV-1-induced cytopathic effect. We chose harmol for further studies because it exhibited the highest antiviral activity. We found that harmol inhibited both HSV-1 F and HSV-1/153 (a clinical drug-resistant strain) replication, with an EC_50_ of 9.34 µM and 5.84 µM, respectively. Moreover, harmol reduced HSV-1 replication in corneal tissues and viral progeny production in tears, and also alleviated early corneal surface lesions related to HSK. For example, harmol treatment preserved corneal thickness and nerve density in HSK mice. Interestingly, harmol also showed a promising antiviral effect on HSV-1/153 induced HSK in mouse model. Furthermore, harmol combined with acyclovir (ACV) treatment showed a greater antiviral effect than either one alone in vitro. Therefore, harmol may be a promising therapeutic agent for managing HSK.

**Supplementary Information:**

The online version contains supplementary material available at 10.1186/s12985-024-02384-0.

## Introduction

Herpes simplex keratitis (HSK) is a prevalent infectious disease in ophthalmology clinics [8] characterized by corneal stromal implication, progressive corneal opacity and visual impairment [34]. The estimated incidence of HSK in developed countries is 10–30/100,000 of the population per year, which is higher in developing nations [38]. In fact, in low-income countries, HSK is responsible for a significant number of cases of blindness compared to other infectious eye diseases [22]. If HSK is not controlled in a timely manner, it can lead to potentially fatal encephalitis [39]. Therefore, exploring HSK treatment options is essential for the clinical management of HSK.

HSK is associated with herpes simplex virus type 1 (HSV-1) infection and lesser extent HSV-2 [9]. HSV-1 is a highly prevalent enveloped double-stranded DNA (dsDNA) virus that exhibits structural complexity and is widely distributed among the global population [4, 41]. Therefore, blocking HSV infection is critical to controlling HSK development. Currently, ACV, a nucleoside analog, has been considered a gold standard medication for HSV infections [43]. However, drug resistance to HSV is on the rise, particularly in immunocompromised patients [36]. Worryingly, current anti-herpesvirus drugs have similar pharmacologic effects [32]. Therefore, it is crucial to explore novel anti-HSV-1 agents that possess unique pharmacological properties.

Harmol, a β-carboline alkaloid, is present in several medicinal plants, including *Peganum harmala, Passiflora incarnate, and Bansteriopsis caapi* [1]. Chemically, harmol (C_12_H_10_N_2_O with Molecular Mass 198.225) is a natural indole alkaloid, 9 H-beta-carboline with a methyl substituent at C-1 and a hydroxy group at C-7; having similar functionality as beta-carboline [35]. Previous study showed that harmol exhibited antifungal effects because harmol suppressed the infection of *Penicillium digitatum* and *Botrytis cinerea* [30]. Furthermore, harmol also inhibited the replication of Newcastle disease virus (NDV, a negative-stranded RNA virus) [44], and dengue virus (DENV, a positive-stranded RNA virus) [33], which suggested that harmol might have potential antiviral activity. The antiviral activity of harmol in HSV (dsDNA virus) infectious diseases, especially HSV-1-related HSK, was largely unknown.

In our current study, we assessed the anti-HSV activity of 502 natural compounds in vitro and harmol was identified as the most effective anti-HSV compound. Then we evaluated the therapeutic efficacy of harmol in the HSK mouse model and found that harmol not only inhibited HSV-1 and HSV-1/153 infections *in* vit*ro* but also alleviated the early symptoms of HSK in vivo.

## Materials & methods

### Cells and viruses

Vero cells were obtained from the American Type Culture Collection (ATCC) and maintained in Dulbecco’s modified Eagle’s medium (DMEM, Gibco) with 10% fetal bovine serum (FBS) (FND500, ExCell Bio, Shanghai, China). HSV-1 F strain (ATCC VR-733) and ACV-resistant clinical HSV-1 strain (HSV-1/153) were kindly provided by Yan Lu, Department of Ophthalmology, Jingling Hospital, Jiangsu, China [17]. HSV-1/153, a TK-mutant derived from HSV-1 (KOS)-HSV-1/Blue was first isolated by Tao Peng (Guangzhou Institutes of Biomedicine and Health, Chinese Academy of Sciences) [45].

HSV-1 and HSV-1/153 were propagated on Vero cells. Vero cells were inoculated with HSV-1 or HSV-1/153 at a multiplicity of infection (MOI) = 0.1 when reaching ~ 80% confluence. And then cultural supernatant containing virus particles was collected when VSV-GFP-infected Vero cells showed > 80% cytopathic effect (CPE). The cultural supernatant containing virus particles was centrifuged at 1000 ~ 1500 g for 5 min to remove cellular debris. Virus particles were preserved in DMEM basic medium at a reduced volume of 1/100 and stored in small aliquots at -80 °C until required. Viral titers were determined by tissue culture infective dose (TCID_50_) assay. Titers were expressed as Log _10_ Plaque-forming unit (PFU) per mL (PFU/mL).

### Regents

The harmol used in this study was isolated from *Peganum harmala* Linn, and was obtained from MedChemExpress (Shanghai, China). Harmol powder was prepared by dissolving it in Dimethyl Sulphoxide (DMSO). ACV was obtained from the National Institutes for Food and Drug Control in China and dissolved in ddH_2_O.The antibodies against gD-1 (Cat# sc-69,802) (Santa Cruz, CA, USA), and GAPDH (CST, MA, USA) were stored at -20℃. Goat anti-rabbit IgG IRDyeTM 680 antibody and Goat anti-mouse IgG IRDyeTM 800 antibody were obtained from LI-COR (Lincoln, NE, USA). RIPA lysis buffer was obtained from Beyotime (Nanjing, Jiangsu, China).

### The natural product library

The library was purchased from the National Center in Shanghai City, which contains 502 natural compounds. Each natural product in the library was highly purified with a known molecular weight and chemical constitution. All of the products were dissolved in 96-well plates with DMSO to 10 mM. The concentration of the compounds used for the screening assay was 10 µM.

### Cytotoxicity assay

Vero cells were treated with harmol or ACV for 72 h, and then cell viability was detected by Cell Counting Kit 8 (CCK-8) (Beyotime Biotechnology, Shanghai, China) assay. Subsequently, OD values were detected at 450 nm. The formula of cell viability was (OD _treatment_ – OD _treatment-blank_)/ (OD _control_ – OD _control-blank_) × 100%. The 50% cytotoxic concentration (CC_50_), the concentration of compound that inhibited the cell viability by 50%, was calculated based on the CalcuSyn computer program [13].

### Natural compounds screening assays

Screening of natural monomer compounds with anti-HSV-1 activity from a library of compounds by a cytopathic effect (CPE) inhibition assay [25]. Vero cells (4 × 10^4^ cells) were seeded into 96-well plates and cultured for 24 h. The cells were treated with each compound (10 µM) in natural product library or ACV (1 µM) at 37 °C for 30 min. ACV was considered a positive control. Subsequently, the cells were infected with HSV-1 F (MOI = 0.5). For the compounds screening experiment, viral inhibition (%) was calculated by CPE. After 48 h cultured, virus-infected Vero cells were placed under an inverted microscope (Nikon, TS100) to observe CPE (%), which was recorded from 0 to 100% depending on the extent of the CPE area [20]. And then the viral inhibition (%) was calculated as follows: 100% - CPE (%). The infected cells showed 100% CPE under the microscope (Scale bar: 50 μm).

### Antiviral activity of harmol

Serial dilutions of harmol were added in 100 µL total volume and infected with HSV-1 (MOI = 0.5) for 48 h. The EC_50_, which represents the concentration of the compound that provided 50% protection against virus-induced CPEs in cells, was determined using the CalcuSyn computer program [13]. Selectivity index (SI) was defined as the safe range for determining the effects of drugs, and SI = CC_50_/EC_50_. Vero cells were pretreated with harmol or ACV, and then they were infected with HSV-1 F or HSV-1/153 (MOI = 0.5 or 1.0) in the presence of the test compound. Subsequently, the in-cell Western assay was used to determine the viral replication or viral progeny by detecting the expression of HSV-1 gD-1 protein. Viral inhibition (%) of harmol was calculated using the following formula: [1 − fluorescence _gD_/fluorescence _control_] * 100%.

### Quantitative real-time PCR (qPCR)

HSV-1F or HSV-1F/153 infected Vero cells were treated with harmol or ACV for 48 h. Total RNA was extracted using Trizol assay, and then reverse transcribed RNA into cDNA with a total reaction volume of 10 µL. Subsequently, 2 × AceQ Universal SYBR qPCR master mix was used for qPCR detection. The qPCR was performed using an ABI 7300 instrument from the USA. The primer sequences of HSV-1 gD-1 were as following : Forward, 5’ AGCAGGGGTTAGGGAGTTG 3’; Reverse, 5’ CCATCTTGAGAGAGGCATC 3’.

### In-cell western assay

The In-cell Western assay was performed according to our previous study [52] via using the Odyssey Imaging System from Li-COR Biosciences (NE, USA). The HSV-1 replication was quantified using Mouse gD-1 antibody (green), and the relative amount of gD protein expression was determined by normalizing it to the endogenous DRAQ5 signal (red) (Biostatus, Cat# DR05500, UK) in all experiments.

### Western blot assay

The cells were lysed by adding RIPA lysis buffer supplemented with protease inhibitors and incubated in an ice bath for 10 min. The total protein concentrations were determined using the BCA protein assay kit from Pierce (Rockford, IL, USA). The western blot assay was performed according to our previous study [52]. The protein bands were scanned using the Li-COR Odyssey Infrared Imager, and then the gray levels of the blots were quantified using Image J software.

### TCID_50_ assay

The viral titers for HSV-1 F and HSV-1/153 were estimated by 50% tissue culture infective dose (TCID_50_) method according to previous methods [23]. Vero cells were seeded in 96-well plates and cultured in DMEM medium supplemented with 2% FBS for 24 h. Subsequently, 10-fold serial dilutions of the supernatant was collected from HSV-1-infected, harmol-treated and ACV-treated Vero cells, as well as HSK mouse tear samples were used to infect Vero cell monolayers for 48 h. Calculations of TCID_50_ were performed according to the *Reed-Muench* method [7], the viral titers were expressed as log_10_ PFU/mL, and PFU/mL = 0.56×TCID_50_ [47].

### Mouse HSK model establishment

To the best of our knowledge, primary infection of the cornea of BALB/c mouse with a large dose of the HSV-1 (1 × 10^5^ PFU/mL) can lead to severe HSK and persist for several months [46]. A previous study found that HSV-1-related corneal infection led to the establishment of sympathetic latency in the HSK mouse model, which was similar to that in HSK patients [21]. Moreover, the pathogenesis of the HSK mouse model was consistent with that of HSK patients [50].

BALB/c mice (*n* = 24) were randomly divided into four groups including control group, HSK group, harmol treatment group, and ACV treatment group. Mice were anesthetized with an intraperitoneal injection of 1% sodium pentobarbital (80 mg/kg) for anesthesia. And then eyes were inoculated with 5 µL of HSV-1 F (1 × 10^5^ PFU/mL), HSV-1/153 (1 × 10^5^ PFU/mL) or PBS by 3 (horizontal) × 3 (vertical) strokes on the right cornea with a 27-gauge needle. 24 h after infection (day 1), mice were treated with harmol (0.01 mg/kg, 5 µL/mouse), ACV (0.01 mg/kg, 5 µL/mouse), or PBS (equal volume of PBS) on days 1, 2, 3, 4 and 5 post-infection via eye drops (3 times/day). Then, we chose days 3 and 5 to evaluate the antiviral activity of harmol in vivo as well as the body weight of HSK mice decreased from day 3 and reached a maximum on day 5, which was consistent with a previous study [49].

### Corneal fluorescein sodium staining and scoring

The mice were treated with fluorescein staining on days 3, 4, and 5. The eyes were then rinsed with 0.9% saline and subjected to a biomicroscope for examination under blue light. The scoring of epithelial keratitis following viral infection (0 to 4) was performed by fluorescein sodium staining based on a previous study [9].

- 0 point: no visible damage.
- 1 point: diffuse punctate lesion.
- 2 point: dendritic lesion occupying less than 1/4 of the entire epithelial area.
- 3 point: severe dendritic lesion extending more than 1/4 of the entire epithelial area.
- 4 point: geographic lesion on the epithelial area.

### Blepharitis scoring

The scoring of blepharitis (0 to 4) on day 3 and 5 post infection (d pi) were determined according to a previous study [28]. The following is a brief description of these criteria:

- 0 point: no swelling of eyelids;
- 1 point: mild swelling of the eyelids;
- 2 points: moderate swelling of the eyelids plus moderate crusting;
- 3 points: half of eyelids shut plus severe crusting;
- 4 points: eyelids completely shut.

### RTvue OCT examination

The corneal thickness was obtained using RTvue OCT (RTvue 100-2, Optovue, Inc, USA), which was equipped with a cornea-anterior module long adaptor lens. The mice were anesthetized and the “pachymetry” scan pattern was used for image collection. To measure the corneal thickness, four points on the horizontal and vertical radial lines within central 1 mm zones of the cornea were selected respectively, and then each point thickness was measured manually. The average thickness of these eight points was calculated as corneal thickness.

### In vivo confocal microscopy (IVCM) examination

BALB/c mice were anaesthetized via intraperitoneal injection with 6 mg/mL ketamine and 4 mg/mL xylazine. The corneas of the mice were positioned on the objective lens and the front surface of a poly-methyl-methacrylate (PMMA) cap (Tomo-cap; Heidelberg Engineering GmbH), which served as a coupling medium, in all IVCM (HRT III RCM, Heidelberg Engineering, Germany) tests. The operator viewed real-time video images displayed on a computer screen, manually adjusting the target area and depth. Subbasal nerve plexus images and corneal vasculature images measuring 400 × 400 microns were then captured. Based on a previous study [12, 16], the images were processed using NeuronJ, version 1.4.3, a plug-in for ImageJ (https://imagescience.org/meijering/software/neuronj/), which traces nerves and calculates the nerve density.

### Statistical analysis

For in vivo experiments, 3–6 mice were included in each group and conducted once experiment in this study. Data were analyzed by GraphPad Prisms version 9.0 software (GraphPad Software, La Jolla, CA, USA). Statistical differences between groups were analyzed by *One-way ANOVA, Repeated measures ANOVA, Dunnett-t test, SNK-q test, Student t test.* Statistical significance was expressed as *P* < 0.05.

## Results

### Harmol inhibits HSV-1 replication

**Fig. 1:**
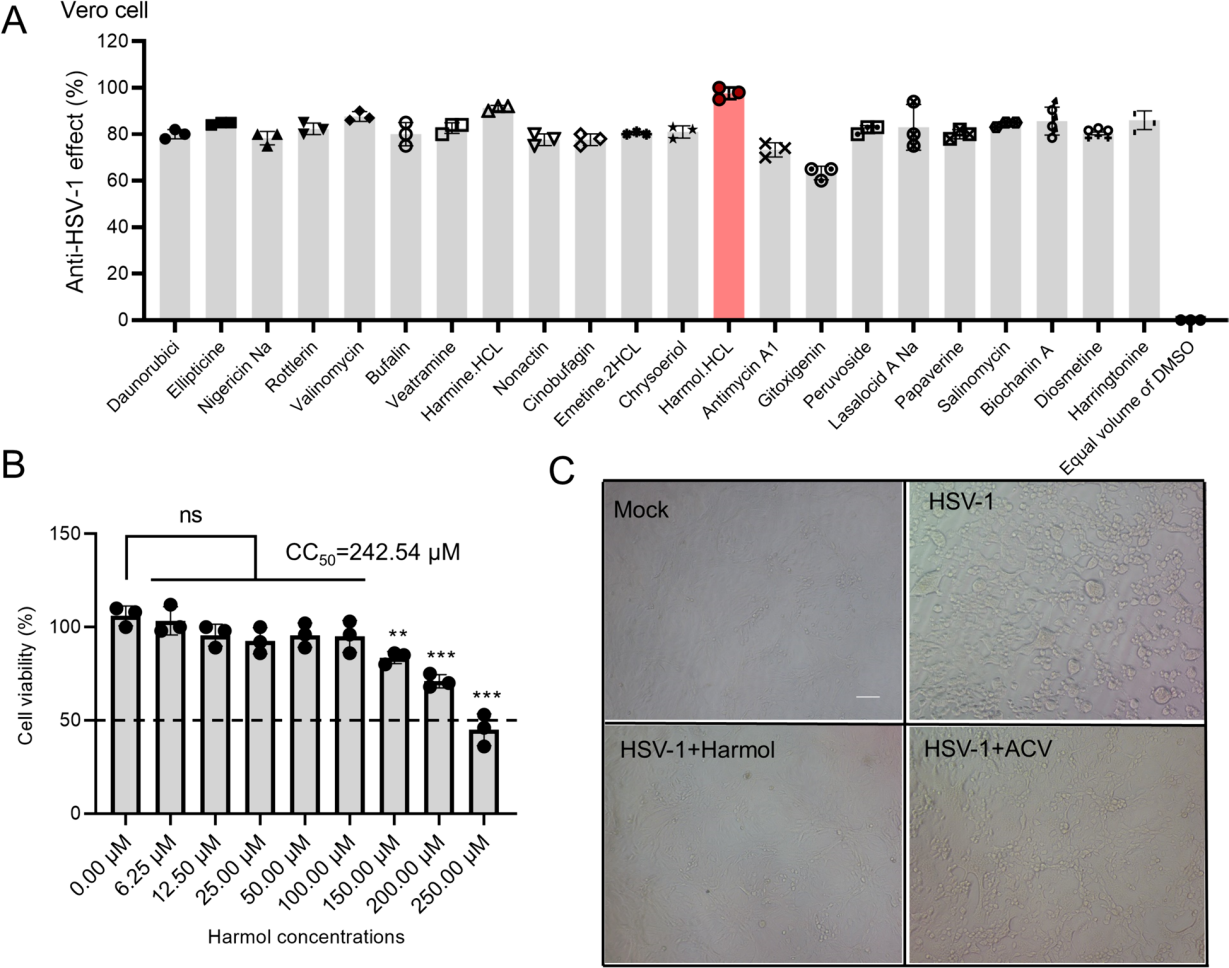
A primary anti-HSV-1 screen assay. **A** After a 2 h pretreatment with these compounds, Vero cells were infected with HSV-1 F (MOI = 0.5). The anti-HSV-1 effect was determined by measuring the CPE as a percentage. **B** Vero cells in 96-well plate were cultured with harmol (0, 6.25, 12.5, 25.0, 50.0, 100.0, 150.0, 200.0, 250.0 µM) for 48 h. Cell viability was measured using a CCK-8 assay. **C** Vero cells were infected with HSV-1 F (MOI = 0.5) and then they were treated with harmol (12.5 µM) or ACV (1 µM) for 24 h. Images were obtained by inverted microscope, scale bar: 50 μm. The experiments were duplicated three times, and corresponding results were shown (*ns*: non-significant, ^**^ *P* < 0.01, ^***^ *P* < 0.001)

We first screened the natural compounds with anti-HSV-1 activity by CPE inhibition assay in Vero cells. We found that 22 compounds showed the percent of viral inhibition > 75%, and harmol (the percent of viral inhibition > 90%) was chosen for further study because of its high antiviral activity (Fig. 1A). We next investigated the cytotoxicity of harmol in Vero cells to explore the proper concentration for further study. In our study, CC_50_ of harmol was 242.54 µM, and non-cytotoxic concentrations of harmol (12.5 µM) were confirmed for the following study in vitro (Fig. 1B). A previous study has demonstrated that the EC_50_ values of ACV for HSV-1 F and HSV-1/153 were 0.209 ± 0.056 µM and > 100 µM, respectively [9]. In our experiment, a working concentration of 1 µM ACV was utilized as a positive control. Both harmol (12.5 µM) and ACV (1 µM) treatments were able to reduce HSV-1-related CPEs (Fig. 1C). In summary, harmol was the most potent anti-HSV-1 agent from the nature product library.

### Harmol inhibited HSV-1 replication in vitro

**Fig. 2:**
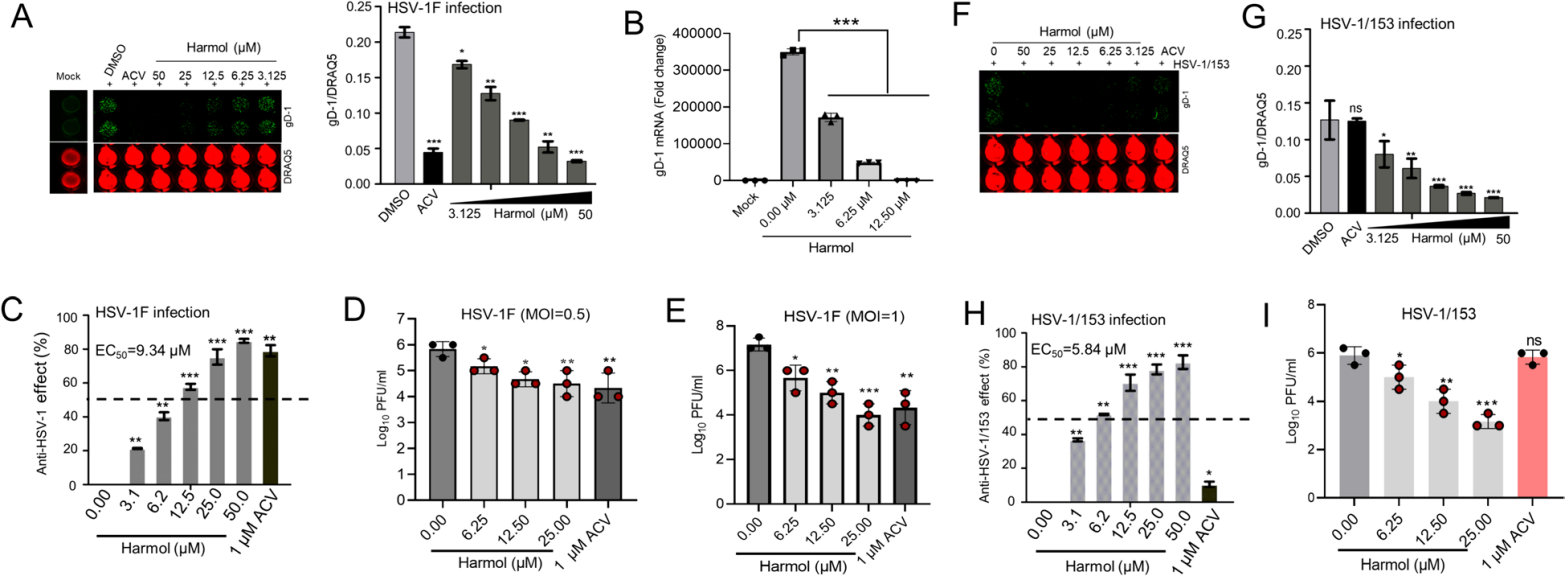
Harmol reduced HSV-1 F and HSV-1/153 replication in vitro. **A** Vero cells were pretreated with ACV (1 µM) or harmol (0, 3.125, 6.25, 12.5, 25, 50 µM) for 2 h, subsequently, they were challenged by HSV-1 F (MOI = 0.5) for 24 h. The HSV-gD-1 (green) expression was identified by in-cell western assay, which was further normalized by DRAQ5 (red). **B** The expression of gD-1 mRNA was detected by qPCR analysis. **C** Anti-HSV-1 effect (%) was counted by in-cell western assay. **D**, **E** HSV-1-infected Vero cells were treated with different concentrations of harmol or ACV. The supernatant was collected at 48 h and the viral titers were measured by TCID_50_ assay. ACV treatment at 1 µM was used as a positive control. All experiments were duplicated three times, and corresponding results were shown. **F**, **G** Vero cells were pretreated with various concentrations of harmol (0, 3.125, 6.25, 12.5, 25, 50 µM) for 2 h, and then they were infected with HSV-1/153 (MOI = 0.5) for 48 h. gD-1 protein expression (green) and DRAQ5 (red) were determined by In-cell Western assay. **H** Anti-HSV-1 effect (%) was shown. **I** The supernatant was collected at 48 h and the viral titers were measured by a TCID_50_ assay. All trials were duplicated three times, and corresponding results were shown. Data expressed as Mean ± SD (*ns*: non-significant, ^**^ *P* < 0.01, ^***^ *P* < 0.001)

Initially, we investigated the anti-HSV-1 effect of harmol by measuring gD-1 protein expression. ACV treatment at 1 µM was considered as a positive control in our present study. ACV (1 µM) and harmol (0, 3.125, 6.25, 12.5, 25, 50 µM) treatments both reduced HSV-1 gD-1 protein expression (Fig. 2A and B). We further assessed the anti-HSV-1 activity of harmol. The EC_50_ of harmol on HSV-1 F was 9.34 µM, which is lower than its cytotoxicity concentrations (Fig. 2C). Harmol could also reduce the production of offspring viruses (Fig. 2D and E). We further attempted to investigate whether harmol could suppress HSV-1/153 replication. Our present results showed that harmol, but not ACV, also reduced the protein level of HSV-1/153 gD-1 in Vero cells (Fig. 2F and G). Furthermore, the EC_50_ of harmol on HSV-1/153 was 5.84 µM (Fig. 2H). Harmol also exhibited a reduction of 2 logs of HSV-1/153 viral titers (Fig. 2I). The SI value of harmol on the HSV-1 F strain was 26.0, and SI value of harmol on the HSV-1/153 strain was 44.5. In summary, harmol reduced HSV-1 F and HSV-1/153 replication and viral progeny production in vitro.

### Harmol, in combination with ACV, showed a better anti-HSV-1 effect

**Fig. 3:**
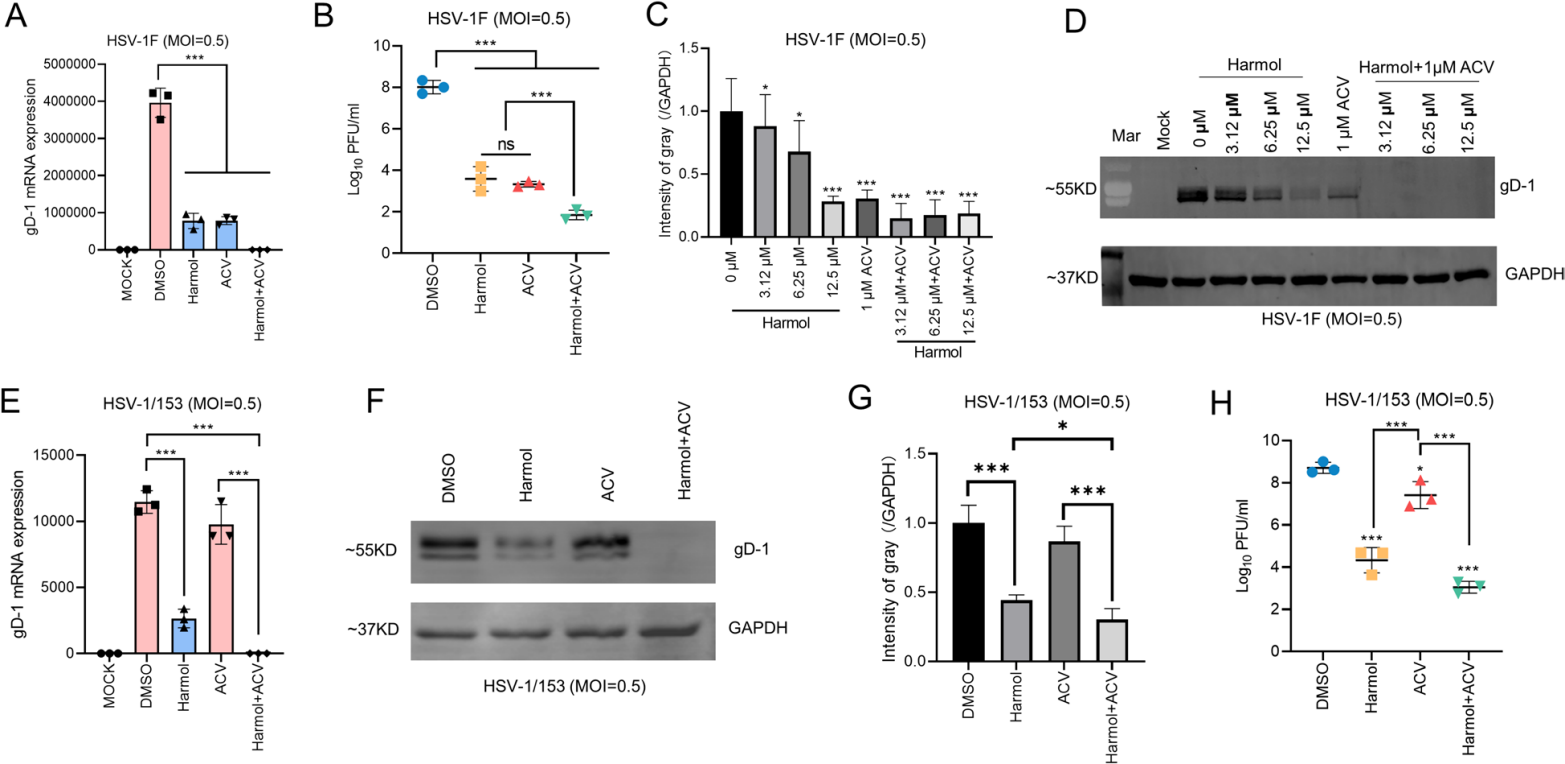
The combination of harmol and ACV was more effective than either one alone. **A** HSV-1 infected Vero cells were treated with harmol (12.5 µM), ACV (1 µM) or harmol (12.5 µM) + ACV (1 µM) for 48 h in the presence of harmol and ACV. qPCR assay was used to detect the gD-1 mRNA expression level. **B** The cell supernatants derived from (**A**), were further evaluated by a TCID_50_ assay, the viral titers were expressed as log_10_ PFU/mL (PFU/mL = 0.56×TCID_50_). **C**, **D** An MOI of 0.5 of HSV-1 F-infected Vero cells were treated with 0.02% DMSO, harmol (0, 3.12, 6.25, 12.5 µM), ACV (1.0 µM) and harmol (3.12, 6.25, 12.5 µM) + ACV (1.0 µM) for 48 h. The gD-1 protein expression was detected by Western Blot assay (**C**) and the quantification of the blots was performed by Image J software (**D**). **E**-**G** HSV-1/153 infected Vero cells were treated with 0.02% DMSO, harmol (12.5 µM), ACV (1.0 µM) and harmol (12.5 µM) together with ACV (1.0 µM). Total RNA and total proteins were harvested to detect gD-1 mRNA and protein expression, respectively. **H** TCID_50_ assay was used to measure the viral proliferation from the cell supernatants derived from (**E**). Data expressed as Mean ± SD (*ns*: non-significant, ^*^ *P* < 0.05, ^***^ *P* < 0.001)

We further assessed the anti-HSV-1 effect of harmol in combination with ACV. Compared to harmol or ACV treatment groups, harmol, together with ACV, inhibited HSV-1 F gD-1 mRNA expression and the production of its progeny viruses (Fig. 3A, B). Furthermore, dose-increased harmol treatment (0, 3.12, 6.25, 12.5 µM) significantly reduced gD protein expression, and harmol in combination with ACV showed better anti-HSV-1 F effects (Fig. 3C, D). The same outcomes were achieved in cells infected with HSV-1/153 (Fig. 3E-H). Altogether, our results indicated that harmol enhanced the anti-HSV-1 effect of ACV, including ACV-resistant strain.

### Harmol may exhibit anti-HSV-1 effects at various stages during HSV-1 infection

**Fig. 4:**
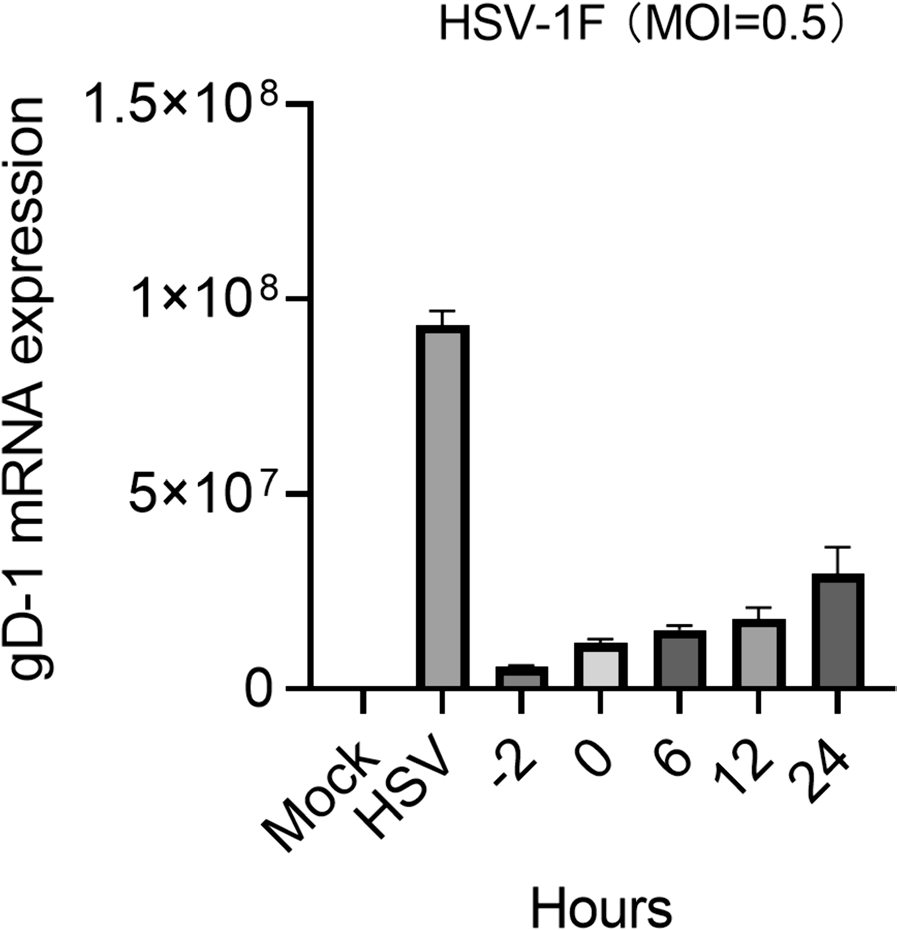
The time of addition experiment of harmol. Vero cells were infected with HSV-1 (MOI = 0.5), and then they were added with harmol (1 µM) at various times (-2 h, 0 h, 6 h, 12 h, and 24 h) post-infection.We utilized qPCR assay to detect gD-1 mRNA expression

To investigate at which stage of the HSV-1 infection cycle harmol exerts its antiviral effects. We next conducted a time of addition experiment. Vero cells were infected with HSV-1 (MOI = 0.5), and then they were added with harmol (1 µM) at various times (-2 h, 0 h, 6 h, 12 h, and 24 h) post-infection. We utilized qPCR assay to detect gD-1 mRNA expression. We observed that pretreatment with harmol significantly inhibited HSV-1 replication, a phenomenon that was also observed at other time points of harmol addition, despite the diminished antiviral efficacy of harmol with prolonged addition time **(**Fig. 4**)**. In conclusion, harmol may exert anti-HSV-1 effects during the early stages of viral infection.

### Safety of topical treatment of harmol on the mouse cornea

**Fig. 5:**
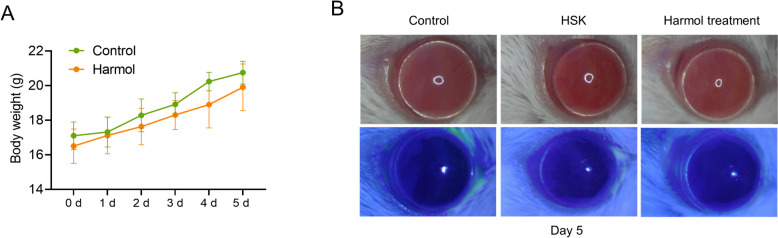
The safety of harmol on the murine cornea. **A** Harmol-treated HSK mice show the weight gain as compared to PBS-treated ones without any significant differences (*n* = 6). **B** Corneal images were collected via white light and cobalt blue light at day 5 (*n* = 6). Data expressed as Mean ± SD (*ns*: non-significant)

To investigate the safety of topical application of harmol on the murine corneas, the eyes were treated with 5 µL harmol (0.01 mg/kg, approximately equal to 100 µM) three times on day 1, day 3, and day 5 as compared to PBS treatment. The body weights of mice were recorded daily with harmol administration, and harmol did not affect the weight of the treated mice compared to those treated with PBS (Fig. 5A). The fluorescein sodium staining confirmed that corneal transparency and epithelial were not damaged by harmol at 5 d (Fig. 5B). Altogether, harmol at a concentration of 0.01 mg/kg had no toxicity to the corneas of mice.

### Eyedrop treatment of harmol alleviated the severity of HSK in vivo

**Fig. 6:**
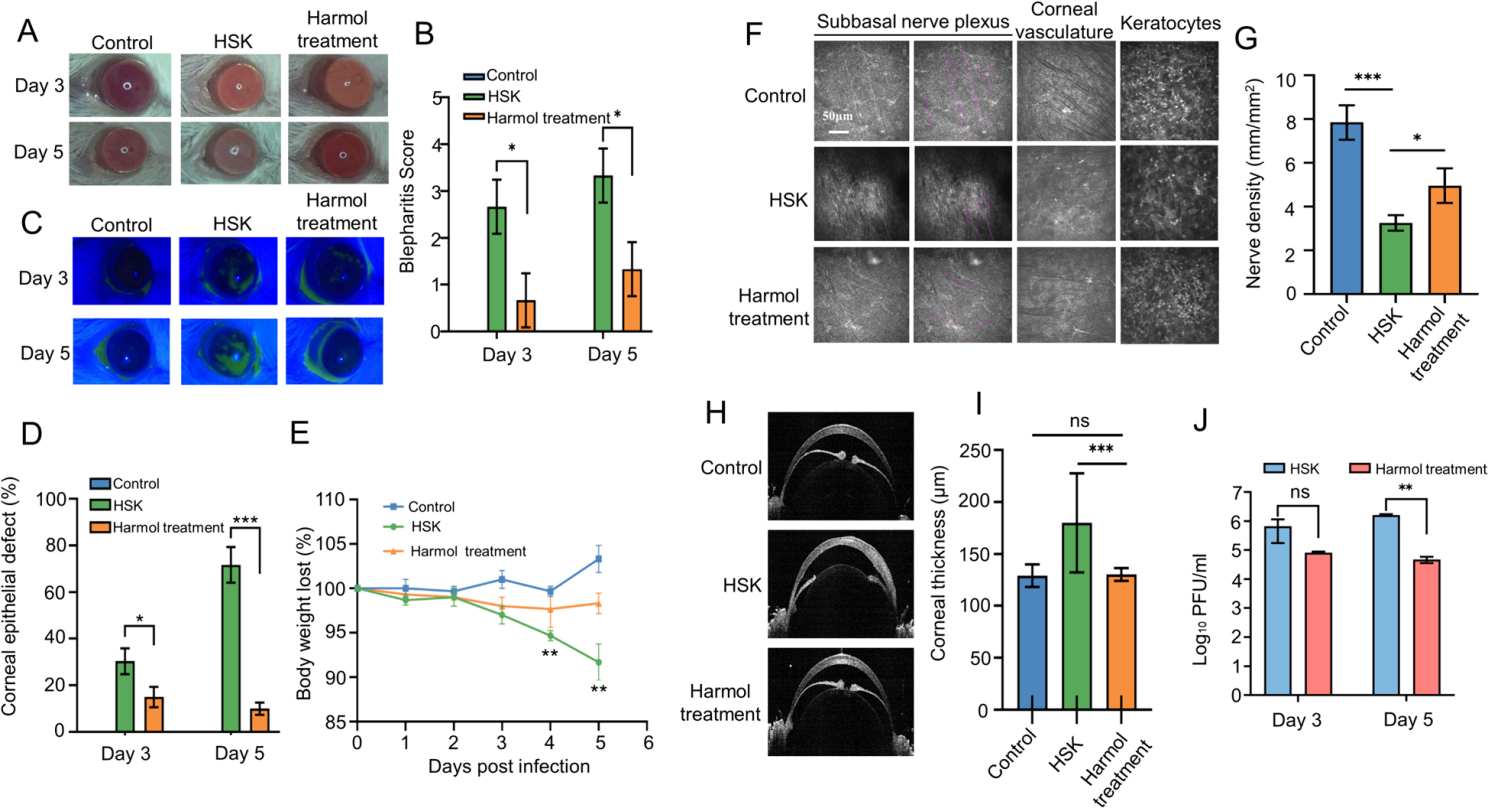
Harmol alleviated the scores of HSK early corneal diseases in vivo. **A** Corneal images at 3 dpi and 5 dpi were obtained following HSV-1 infection. **B** Blepharitis scores were calculated at 3 dpi and 5 dpi (*n* = 6). **C** Photographs of sodium fluorescein staining were recorded in mice (*n* = 6). **D** The relative defect (%) of corneal lesions was calculated as percentage of fluorescein staining areas to the whole corneal. **E** Rate of weight loss in mice infected with HSV-1. **F** Representative images of subbasal nerve plexus, corneal vasculature, and keratocytes at 5 dpi using IVCM. Traced nerve fibers are observed in purple. **G** Corneal nerve density was quantified by Image J (*n* = 6). **H** Corneal thickness was obtained by RTvue OCT at 5 dpi. **I** Corneal thickness was measured. **J** The tear swabs of HSK and harmol treatment groups were calculated via TCID_50_ assay (*n* = 6). Data expressed as Mean ± SD (*ns*: non-significant, ^*^ *P* < 0.05, ^**^ *P* < 0.01, ^***^ *P* < 0.001)

Next, we established an HSK mouse model to assess the anti-HSV effect of harmol in vivo. Compared to the HSK group, harmol treatment relieved HSV-1 F-related corneal opacity at 3 and 5 dpi (Fig. 6A). Blepharitis was observed in the HSK group at 3 and 5dpi, which was alleviated by harmol treatment due to a reduction in blepharitis scores (Fig. 6B). Corneal fluorescein staining with cobalt blue light was used to assess corneal epithelial injury, and harmol treatment also alleviated HSV-1 F-induced early corneal injury (Fig. 6C). In addition, harmol-treated mice showed less fluorescein staining and there was no epithelial defect in uninfected corneas (Fig. 6D). Furthermore, harmol treatment significantly reduced the body weight loss caused by HSV-1 F infection (Fig. 6E). All together, these results demonstrated that topical application of harmol efficiently ameliorated the ocular diseases in mice with HSV-1 infection.

SNP density, corneal vasculature, and keratocytes of HSK mice were observed by IVCM after 5 days of infection. The central nerve density in HSK group was notably reduced as compared to the control group (3.26 ± 0.36 vs. 7.85 ± 0.78 mm/mm^2^); HSV infection reduced the central nerve density was significantly alleviated in the harmol treatment group (4.96 ± 0.79 mm/mm^2^) (Fig. 6F and G). We were not able to evaluate the effect on corneal neovascularization because we only scored the disease process on day 5 post-infection as well as the acute HSK model was well performed as long as on day 7 post-infection. Furthermore, we observed that harmol treatment significantly alleviated HSK-related early corneal injury, such as corneal vascular damage, disruption of stromal architecture, disorganized collagen lamellae, scattered microdeposits, and reduced keratocytes (Fig. 6F). Moreover, RTvue OCT was used to examine the corneal thickness of mice at 5 dpi. As shown in Fig. 6H and I, harmol-treated mice retained ocular structural integrity as compared with HSK mice; however, the limbal epithelium and surrounding conjunctiva of HSK mice were significantly thickened (179.80 ± 47.70 vs. 130.30 ± 6.17 μm). The tear swabs from harmol and PBS-treated groups were collected at 3 dpi and 5 dpi and the viral titers were calculated by TCID_50_. As expected, harmol treatment decreased 2 logs of HSV-1 F viral titers in tears at 5 dpi (Fig. 6J). Our results demonstrated that topical application of harmol not only inhibition HSV-1 replication in vivo but also alleviated HSV-1 F-induced early ocular pathology.

### Harmol alleviated HSV-1/153 induced HSK early corneal diseases

**Fig. 7:**
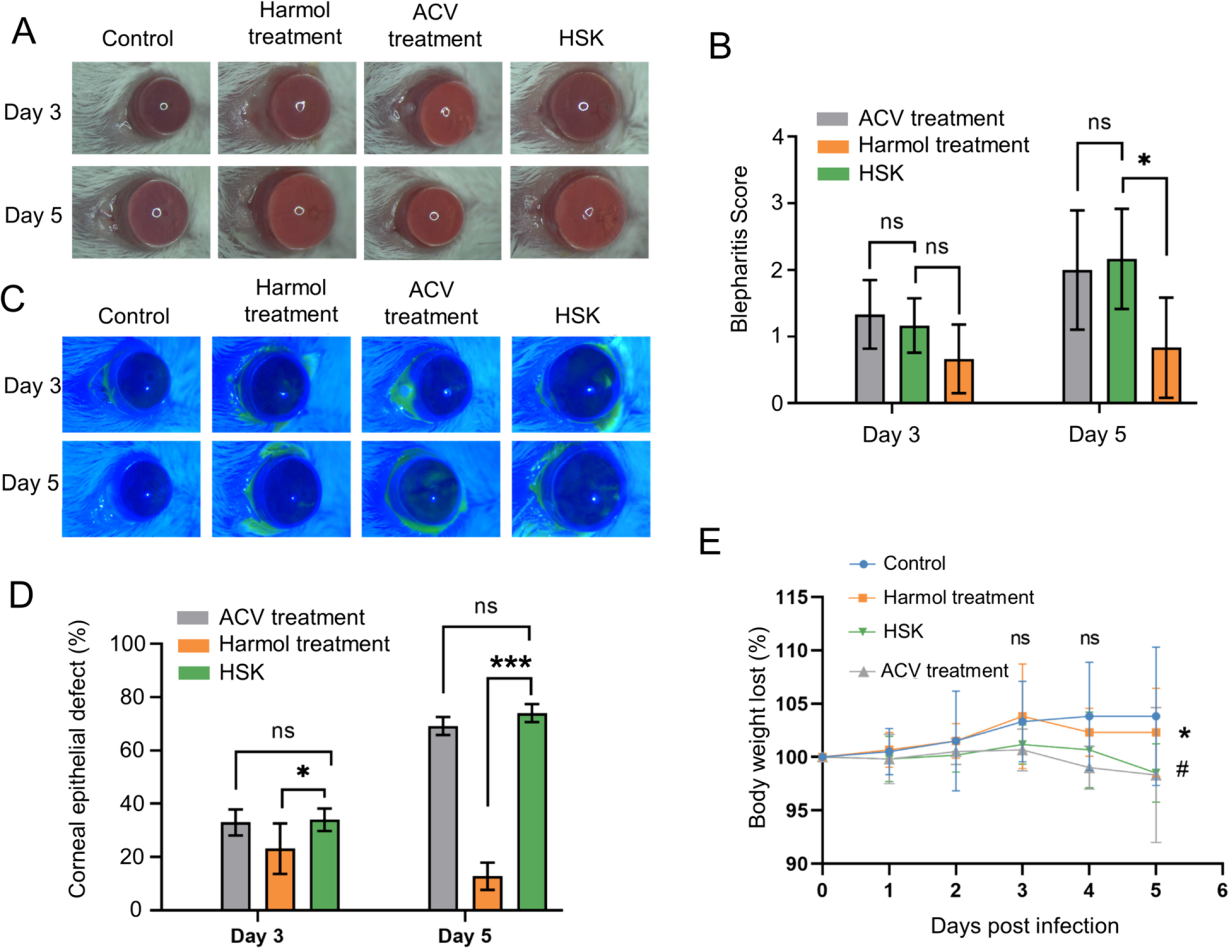
Harmol not ACV alleviated HSV-1/153-induced early HSK corneal diseases. **A** Corneal images at 3 and 5 dpi were obtained following mice with HSV-1/153 infection. **B** Blepharitis scores were calculated at 3 and 5 dpi (*n* = 6). **C** Photographs of sodium fluorescein staining were recorded in mice (*n* = 6). **D** The relative defect (%) of corneal lesions were calculated as percentage of fluorescein staining areas to the whole corneal areas. **E** The relative weight of mice for 5 days after infection (*n* = 6). Data expressed as Mean ± SD (*ns*: non-significant, ^*^ *P* < 0.05, ^**^ *P* < 0.01, ^***^ *P* < 0.001)

In the present decades, the emergence of HSV-1 resistance to ACV has been increasingly reported [5]. HSV-1/153, an ACV resistance strain, was used for further in vivo study. We found that harmol not ACV treatment significantly reduced the blepharitis scores at 5 dpi in mice with HSV-1/153 infection (Fig. 7A and B). Furthermore, harmol treatment notably alleviated the reduction of the early defect of corneal lesions caused by HSV-1/153 infection at 5 dpi (Fig. 7C and D). In addition, we also observed that harmol treatment protected mice from weight loss caused by HSV-1/153 infection; however, ACV treatment could not protect HSV-1/153-infected mice from weight loss (Fig. 7E). Collectively, harmol could fight against ACV-resistant HSV infections in vivo.

## Discussion

The development of HSK was associated with HSV-1 infection, which has led to vision loss and even blindness in patients worldwide. As a result of the overuse of medications, drug resistance in HSV infectious disease has significantly risen, presenting a major public health challenge [42], which is one of the key factors in the protracted course of HSK. Thus, new drugs with different mechanisms of action and minimal toxicity are needed for the treatment of HSV infectious diseases [27]. At present, harmol, a β-carboline alkaloid, not only inhibited HSV-1 F and HSV-1/153 strain replication in corneal tissues of mice but also alleviated viral early infection-induced corneal lesions.

Harmol has also been identified as an antiviral agent for the management of RNA viruses, including NDV and DENV [33, 44]. At present, harmol treatment notably suppressed HSV-1 F infection in vitro and in vivo. Interestingly, the replication of HSV-1/153, the ACV-resistant strain, was also suppressed by harmol but not ACV treatment in vitro and in vivo. Nucleoside analogs represented by ACV are the main drugs for HSV-1 infectious disease management [27]. ACV inhibited viral replication by interfering with viral DNA polymerase activity and incorporating viral DNA [36]. A previous study reported that the resistance of HSV to ACV was linked to mutations in viral DNA [37]. At present, harmol, in combination with ACV, showed a better anti-HSV-1 effect, which indicated that harmol exhibited anti-HSV-1 activity was different from ACV. Interestingly, this study found that the time addition of harmol significantly inhibited the expression of HSV-1 gD-1 mRNA at 24 h post-infection, suggesting that harmol may inhibit HSV-1 replication at various stages of the replication process **(**Fig. 4**)**. A previous study reported that β-Carbolines, including 9-methyl-norharmane, 9-methyl-harmane, and 6-methoxy-harmane, whose structures were similar to harmol, were identified as novel antiviral agents against HSV-1 and HSV-2 [15]. Furthermore, Bag et al., found that harmaline, one of the β-Carbolines, interfered with the binding of the immediate-early complex to the ICP0 promoter during the immediate-early stage of viral replication [6]. Additionally, Gonzalez et al., also reported that β-Carbolines were found to interfere with the late proteins expression [15]. Our study also suggested that harmol, a β-Carbolines compound, may affect HSV-1 replication at various stages of the viral replication process, since it exhibited significant antiviral activity from 0 to 24 h post-infection. In summary, we induced that the antiviral mechanisms of harmol may be different from ACV treatment because harmol blocked the HSV-1 replication even when it was added at late time during HSV-1 infection.

In addition to the possible antiviral mechanisms of β-Carbolines, there are multiple potential antiviral mechanisms of harmol based on previous studies. A previous study have identified targeting the autophagy process as an antiviral pathway to affect the viral replication [19]. The autophagy activation inhibited HSV-1 replication [24, 51], which was associated with the activation of antiviral innate immune response [26, 51]. Harmol has been reported to be an autophagy activator in a variety of disease models. For instance, harmol can activate the autophagy process by triggering extracellular signal-regulated kinase 1/2 (ERK1/2) and AMP-activated protein kinase [3, 48]. Previous studies have reported that the activation of ERK or AMPK pathways were important for the host fighting against HSV infection [14, 18]. Furthermore, Deyan Chen et al., reported that harmine, also known as β-carboline alkaloid, blocked HSV-1 infection through inhibiting NF-κB and MAPK pathways in vitro [10]. Subsequently, they also found that harmine suppressed EV71 replication via inhibiting the NF-κB pathway in vitro [11]. The above evidence implied that harmol might also suppress HSV-1 F or HSV-1/153 replication and alleviate pathologic damage of HSK through targeting NF-κB and MAPK pathways. Harmol has been identified as one of the metabolites of harmine [2], and harmol has been demonstrated as a better therapeutic window as compared to harmine in multiple disease models [31, 40]. Furthermore, the toxic effects of harmol were lower than harmine [29]. Therefore, we speculated that harmol also probably exerted an antiviral effect via modulating the autophagy pathways or NF-κB pathways, which has the potential to address the problem of ACV resistance in HSV-1 in the future, and further studies will be required to identify the exact mechanism of the antiviral effect of harmol.

However, there are some limitations in this study. The primary objective of this study was to assess the antiviral effect of harmol. As a result, we only examined the pathological alterations in HSK at 3 and 5 dpi. It should be noted that this limited timeframe of HSK acute model may not provide a comprehensive evaluation of corneal clouding or corneal neovascularization. Furthermore, the evaluation of harmol’s anti-HSV-1 efficacy in this study is preliminary, therefore, these results should be interpreted with caution.

## Conclusions

These findings demonstrated that harmol induced a significant resistance to HSV-1 F and HSV-1/153 replication in vitro and in vivo and alleviated early symptoms of HSK. Thus, harmol was a promising new anti-HSV-1 agent with a different action from ACV for the new anti-HSV-1 therapy.

### Supplementary Information

Supplementary Material 1.

## Data Availability

No datasets were generated or analysed during the current study.
