# Supplementary figures and images for: Harmol used for the treatment of herpes simplex virus induced keratitis
Source: Virol J. 2024 May 27;21:118. doi: 10.1186/s12985-024-02384-0 (PMC11131330; doi:10.1186/s12985-024-02384-0)

Figure 3D

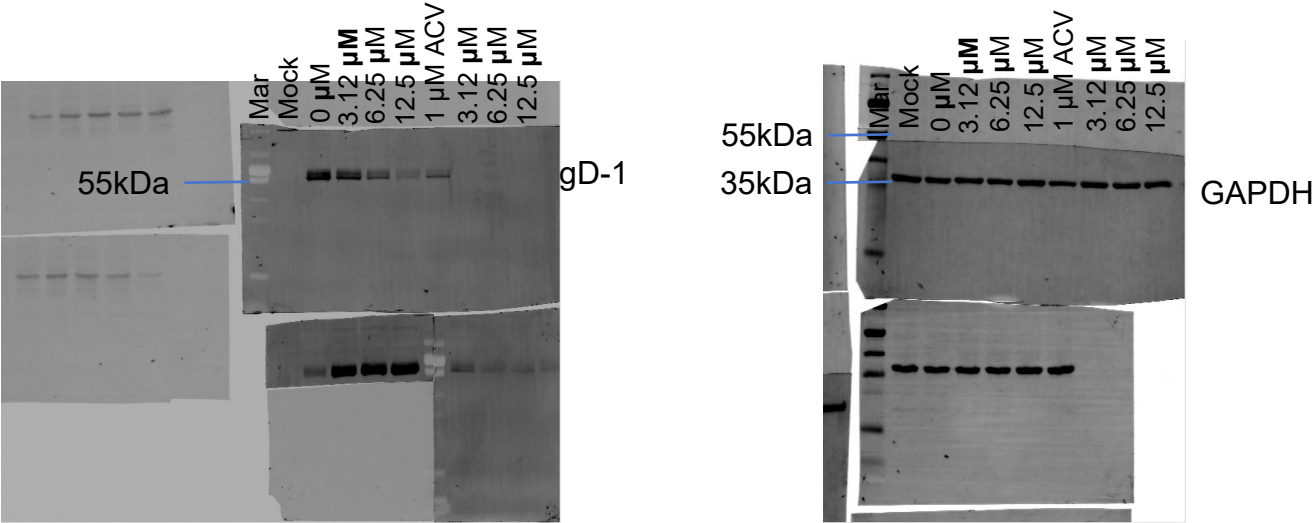

Figure 3F

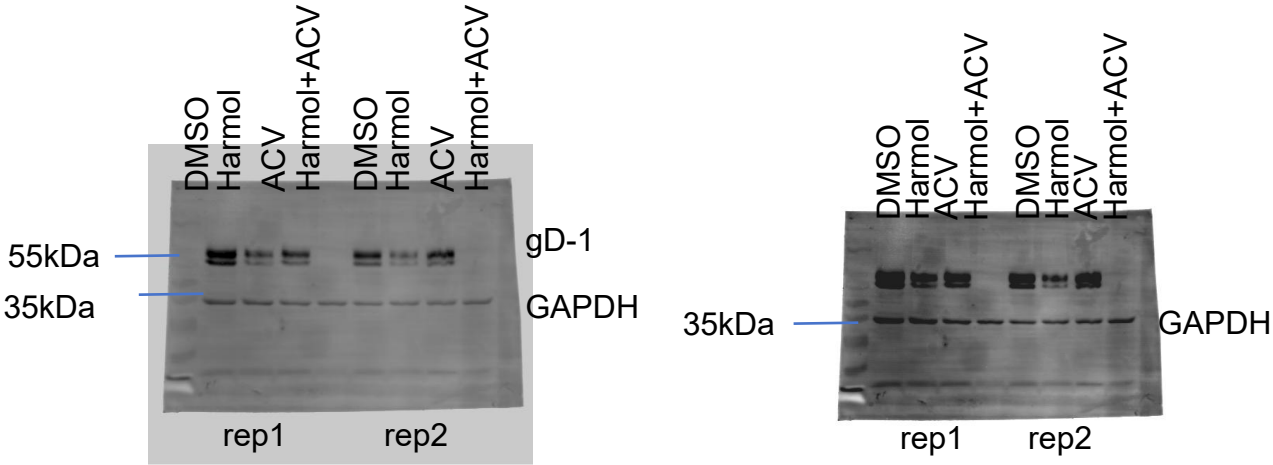

Supplement: Supplementary file 1 — Supplementary Material 1. [file 12985_2024_2384_MOESM1_ESM.pdf]
